# Keyword Index for Volume 105

**DOI:** 10.1038/bjc.2011.540

**Published:** 2011-12-06

2D: 4D 438

2-hydroxyestrone 1458

27.12 MHz 640

3′UTR 296

4-methylumbelliferone 1839

5-azacytidine 1533

5-fluorouracil 1322, 1654

5-fluorouracil/folinic acid 58

14q32.31 locus 1352

16*α*-hydroxyestrone, post-menopause 1458

*α*-smooth muscle actin 1224

*α*_v_ integrins 346

ABCB1 513

aberrant genetic markers 89

acquired resistance 382

activin A 1210

acute lymphoblastic leukaemia 1409

acute myeloid leukaemia 1927

adenocarcinoma 673

adenocarcinoma/adenosquamous carcinoma 420

adenovirus 1302

adiponectin 562

*ADIPOQ* 562

adjuvant hormonal therapy 1628

adjuvant treatment 1480

advanced cancer 945

aerobic glycolysis 469

aetiology 481, 911

afatinib 1554

affluence 1783

African Americans 1096

age 18, 1788, 1814

age–period–cohort models 1795

AKT 329

alcohol consumption 1089, 1430

ALDH 212

aminolevulinic acid 961

anal cancer 498, 1719

androgen deprivation therapy 1628

androgen receptor 1676

androgens 709

aneuploidy 1218

angiogenesis 9, 74, 112, 139, 1002, 1210, 1856

Angiopep-2 1697

angiopoietin 975

angiostatin 1750

angiotensin II type 1 receptor 1331

anthracyclines 1663

antiangiogenesis 44

anti-EGFR therapy 255

ANXA10 1379

anxiety 1814

*Apc*^min/+^ mouse 649

Apo2L/TRAIL 1830

apoptosis 221, 407, 428, 565, 1012, 1302, 1313, 1542

apparently stable patients 612

ARMS/S 403

aromatase inhibitors 1825

Asia-Pacific 945

aspirin 1107, 1776

assessment 628, 903

association studies 864, 1934

ataxia telangiectasia 586

ATM 586

ATM kinase 586

ATR 372

atrial fibrillation 881

attributable proportion 731

autologous 970

awareness 18, 925, 1474

Bangladeshi 925

Barrett’s oesophagus 200, 1218

Bayesian trials 599

BCG 687

bead array 1574

benefit finding 1158

benzo(a)pyrene 1096

bevacizumab 1693

biliary stones 1424

biliary tract cancers 1370, 1424

biomarkers 104, 112, 139, 272, 353, 565, 1379, 1733, 1830, 1894

birth cohorts 177

Birt–Hogg–Dubé syndrome 1912

bladder cancer 393, 1379

bleomycin 766

blood pressure 1693

blood vessel 698

blood–brain barrier 1697

body mass index 162, 1061

body size 1061

bone metastasis 1805

Bowen’s disease 824

brain cancer 1697

brain natriuretic peptide 1663

brain tumour 1414

BRCA1 1114

BRCA2 1114

*BRCA2* gene 1230

breast 723

breast cancer 9, 13, 18, 22, 189, 272, 366, 452, 534, 542, 586, 709, 847, 911, 953, 1082, 1089, 1197, 1224, 1388, 1443, 1451, 1474, 1574, 1669, 1676, 1825

breast cancer biomarker 1203

breast cancer risk 731

breast ductal carcinoma *in situ* 1203

breath condensate 1183

Breslow 1726

brivanib 44

bronchial brushing 1183

cachexia 1244

cadherin switch 1885

cancer cachexia 83

cancer incidence 460

cancer phenotype 1697

cancer progression 1885

cancer registration 1795

cancer registries 170

cancer risk 1230

cancer screening 475

cancer stem cells 212, 1253, 1759

cancer survivors 1158

cancer treatment 1295

cancer type 1814

cancer-associated fibroblasts 996, 1224

cancer-free survival 460

capecitabine 58, 206, 505, 1273

carboplatin 897

carcinoma 694, 1396

cardiac biomarkers 1663

cardiotoxicity 1663

caspase 1830

Cathepsin S 1487

CD133 212, 658

CD34 970

CD44 658

CD45RO 1191

CD9P-1 1002

Cdc42 1313

CEA 239

cediranib 884

cell cycle 552

CellSearch 847

cellular inhibitor of apoptosis protein 2 1322

cervical cancer 177, 420, 486

cervical cytology 983

cervical screening 177

cervix 28, 337, 694, 723

cetuximab 44, 206

chemoradiotherapy 498

chemoresistance 1522, 1759

chemosensitisation 372

chemotherapy 221, 746, 766, 1123, 1166, 1260, 1759

chemotherapy resistance 773

chemotherapy-induced anaemia 1267

childhood cancer 606, 1392, 1402

childhood leukaemia 1783

Chinese 1430

Chinese women 1089

chlamydia 602

cholangiocarcinoma 131, 1885

chromogranin A 1173

chromosome 20q 281

chronic lymphocytic leukaemia 221, 1076

CIP2A 989, 1905

circulating tumour markers 239

circulating endothelial cells 112

circulating tumour cells 847, 1338

cisplatin 1273, 1331

clear cell renal cell carcinoma 1741

clinical 628

clinical outcome 1203

clinical protocol 1166

clinical stage I 493

clinical toxicities 1811

clinical trial 778, 1260

c-Met 131

c-Myc 146, 1563

CNTO 95 346

coffee 157

cohort study 438, 911, 1436

cold plasma 1295

colon cancer 212

colorectal cancer 53, 58, 162, 206, 239, 255, 281, 403, 452, 475, 552, 562, 666, 870, 1039, 1158, 1253, 1346, 1487, 1495, 1759

colorectal tumour 246

combination therapy 353, 1522

communication 918

co-morbidities 1123

comparison 1788

complex intervention 18

conditioned medium 1759

congenital anomalies 1392

Correa pathway 658

cost effectiveness 1273

cotton 1054

COX-2 13

COX2 inhibitors 393, 452

CpG oligodeoxynucleotides 1533

CRIPTO-1 1030

critical period 1443

cross-sectional study 1474

c-Src 953

CTGF 231

culture 918

curcumin analogue 212

cutaneous squamous cell carcinoma 824

cyclooxygenase inhibition 1107

Cyclooxygenase-2 13, 534

cytokines 687

cytological screening 337

cytostatic 682

cytotoxic 682

dacarbazine 346, 353

dasatinib 118

DASL assay 1574

DCIS 13

deaths 1788

decision making, computer assisted 1166

delayed infection 1783

delayed presentation 18, 1474

delta133p53 1593

dendritic cells 413, 787, 961

depression 1814

deprivation 1783

descriptive epidemiology 1684

D-Glucuronyl C5-epimerase 74

diagnosis 1600

diarrhoea 1654

dideoxy sequencing 246

dietary cadmium exposure 441

digit ratio 438

diphosphonates 881

direct sequencing 403

discoid fibroma 1912

disease stage 1814

disease-free survival 1288

disease-specific survival 1864

DNA 239

DNA methylation 312

DNA repair 773, 1114

DNA repair genes 1542

docetaxel 366, 505, 1480

dose escalation 1646

dose-dense regimen 1480

dose-density 766

dose–response 505

drug 1697

drug response 1708

dulanermin 1830

dysplasia 200, 1218, 1582

E2F 1719

E3 ligase 428

early 628

early breast cancer 1480

early detection of cancer 340

EBV 38

E-cadherin 393

EGFR 1, 420, 523, 760, 938, 1850, 1920

EGFR inhibitor 1522

EGFR pathway 1542

eIF4E 329

elderly 189, 1123

elderly breast cancer 1260

electromagnetic fields 1409

EMT 382, 393

endometrial cancer 1458

endostatin 1615

endotoxin 1054

enterolactone 1151

EpCAM 312

epidemiology 154, 200, 731, 746, 1414, 1419, 1768, 1776

epidermal growth factor receptor 131, 618, 649

epigenetics 1533

epithelial ovarian cancer 441, 1288

epithelial-mesenchymal transition 1338

epithelial-to-mesenchymal transition 1885

epothilone B 1646

Epstein–Barr virus 320

ER81 124

ErbB-2 796

ErbB3 523

ErbB3 antibody 523

erlotinib 1, 382, 760, 938, 1554, 1850

erythropoietin 1267

ethics 918

ethnic 486, 918

ethnic group 1474

ethnicity 481, 1049

ETV1 124

ETV4 124

everolimus 1635

exercise 1443

expression 1346

extracellular matrix 1839

fatigue 445

FDG-PET 498

febrile neutropaenia 606, 612

fertility-sparing surgery 1288

fibre in diet 1151

fibroblast growth factor receptor 1 1362

fibrofolliculoma 1912

FISH 89

fish oil 1469

FOLFOX 760

folliculin 1912

formalin-fixed, paraffin-embedded (FFPE) 1574

formalin-fixed, paraffin-embedded samples 1362

formalin-fixed paraffin-embedded specimen 403

G2 checkpoint 372

gall bladder 154

gamma delta T cells 778

gastric cancer 38, 124, 505, 658, 1210, 1273, 1522, 1750

gastrointestinal tumours 44

gefitinib 1, 382, 1131, 1920

gender 1814

gene amplification 420

gene copy number 1

gene expression analysis 1362

gene expression profiling 304

gene signature 1600

gene therapy 1302

genetic 1244

genetic alteration 1927

genetic association study 870

genetic predisposition to disease 870

genetic susceptibility 1934

genomic profile 1940

genotype 1676

geriatric oncology 189

germ cell tumours 575, 766, 854

germinoma 575

glioblastoma 1235

glioma 961

glutathione S-transferase Pi 1224

GnRH antagonists 1628

gonorrhoea 602

gossypol 221

grade 989, 1288

growth factors 766

growth kinetics 682

GS-168AT2 1002

GSI 796

GSK3*β* 1313

guidance manual 194

gynaecological cancer survivors 737

H_2_-receptor antagonists 1173

H69 814

haematological cancers 452

hazard ratios 157

HBx 146

head and neck cancers 1419

health inequality 1039

health knowledge, attitudes, practice 340

health-related quality of life 606

heparan sulphate proteoglycan 74

hepatocellular carcinoma 146, 640, 945, 1430

HER2 366

Her2/neu 1176

Her2/neu extracellular domain (ECD) 1176

heterocyclic amines 1096

heterodimerisation 807

heterogeneity 139

HGF 814

HIF-1*α* 953

histone modifications 312

histopathological examination 1197

HIV-protease inhibitors 513

HLA1 687

HNSCC 1864

Hodgkin lymphoma 1776

homeobox 565

homologous recombination 372, 1114

hormonal therapy 945

hormone receptor 1089

hormone-naive and castrate-resistant prostate cancer 1362

hormones 709, 1451

HOXC11 118

HOXC8 288

HPV 28, 1719

HPV test 694

HPV vaccination 177, 486

HRT 22

HSP-70 961

HS-SPME/GC-qMS 1894

human herpes virus 8 1768

human papilloma virus 337, 983, 1183

hyaluronan 1839

hypotension 1693

hypoxia 231

IAC 1370

ICON6 884

ICR62 1554

IFN-*λ* 1302

ifosfamide 897

IL-12 1533

IL-6 407

image cytometry 1218

immunohistochemistry 1, 131, 255, 673, 1342

immunotherapy 687, 778, 787

immunovisibility 687

incidence 1049, 1069, 1414, 1419, 1684, 1795

incidence rate 481

incidence trends 177

incompleteness 170

India 723

infants 1940

insulin resistance 1424

insulin-like 1 growth factor receptor 649

interaction 996

interleukin-6 1370

international 1788

interpreter 918

intestine 1253

intetumumab 346

invasion 231, 534, 1030, 1582, 1750

invasive ductal carcinoma 698, 1203

irinotecan 53, 1131, 1522

ISET 847, 1338

isoforms 854

Jagged1 1805

JAK 407

Kaposi’s sarcoma 513

Kaposi’s sarcoma-associated herpes virus 1768

Ki 67 expression 1342

Ki67 272

kidney cancer 1096, 1741, 1772

K-Ras 246

*KRAS* 281, 403

laminin 5-*γ*2 824

lapatinib 618

LEC motility 263

*let-7* 296

letrozole 1825

leucine-rich *α*-2-glycoprotein 1370

leukaemia 1402, 1684

leukopenia 360

lifetime risk 460

linear discriminant analysis 1600

linoleic acid 1750

liver cancer 154

liver metastasis 281, 288

lomeguatrib 773

long-term survivors 1158

long-term side-effects 737

low-density lipoprotein receptor-related protein 1 (LRP1) 1697

luminal B 272

lung cancer 320, 673, 807, 847, 1002, 1042, 1049, 1054, 1123, 1183, 1608, 1850, 1920

lung metastasis 1839

lymph node 698

lymph vessel 698

lymphadenectomy 493

lymphokine-activated killer cells 787

lymphoma 586, 1402, 1414, 1684, 1768

M30 1830

malignant germ cell tumour 493

malignant melanoma 231, 1023

malignant mixed Mullerian tumours 897

mammography 1082, 1669

marker 673, 1726

matrix metalloproteinase-13 1615

maxillary sinus 833

Mcl-1 221

meat intake 1096

medical oncology 1166

megestrol acetate 945

MEK 382

melanin pigmentation 1874

melanocytic proliferations 1023

melanoma 118, 346, 353, 565, 773, 787, 1030, 1076, 1396, 1615, 1726, 1874

memory T cell 1191

menopause 22

mental health 445

mesothelioma 1542

MET 407, 814

*MET* amplification 807

meta-analysis 93, 1451

metabolic reprogramming 469

metabolic response 498

metabolic syndrome 1424

metastasis 74, 1338, 1379, 1615, 1741, 1905

metastatic colon cancer 1646

metastatic non-small cell lung cancer 1338

methodology, trial 194

methylated and unmethylated sequence 65

methylation 575, 1927

methylation sensitive restriction endonuclease-qPCR 65

MGMT pseudosubstrate 773

MHC class I downregulation 1533

MICB 13

microarray 320

microcalcifications 1669

microenvironment 996

micrometastasis 1197

microRNA 104, 320, 833, 1023, 1352, 1733, 1741

microtubule stabiliser 1646

middle aged 157

migration 1615

migration and invasion 407

*mir-101* 296

miR-15b 1719

miR-16 family 146

miR-215 1741

*miR-874* 833

miRNAs 146

mismatch repair gene 162

MMP1 124

molecular epidemiology 65

morbidity 1279

mortality 1076, 1082, 1388

MRI 139

mRNA expression 854

MSX2 565

MTHFR polymorphisms 1654

mTOR 329, 953

mucositis 1654

multidrug resistance 513

multi-kinase inhibitor 1640

multiple myeloma 1708

Musashi-1 658

muscle loss 1469

muscle protein degradation 83

mutation 1, 246, 814

mutation screening 1230

*MYCN* 296

myelodysplastic syndromes 975

myeloma 970, 1684

myofibroblasts 996

n-3 fatty acids 1469

natural killer cells 787

needle biopsy 1600

neoadjuvant chemoradiation 1654

neoadjuvant chemotherapy 9

neoadjuvant hormonal therapy 1628

neoplasms 340, 445, 723, 881, 1934

nested case–control study 1458

neuroblastoma 89, 296, 1352, 1940

neuroendocrine tumours 1173

neuroma 1069

neuropathy 360

NF-*κ*B 263, 1012, 1874

N-myc 296

node location 1137

node ratio 1137

nomograms 599, 1144

non-chlamydial, non-gonococcal urethritis 602

non-*MYCN*-amplified 1352

non-sentinel lymph node metastasis 1197

non-small cell lung cancer 938, 1131

non-steroidal anti-inflammatory drugs 1776

Notch signalling 1805

Notch-1 796

Nottingham Prognostic Index 698

novel radiosensitisers 628

N-terminal pro-brain natriuretic peptide 1663

nucleotyping 1218

nulliparity 731

Nutlin-3a 1012

obesity 1061

occupational 1443

OCT3/4 854

oesophageal adenocarcinoma 200

oesophageal cancer 104, 842, 1302

oesophagogastric cancer 760

oestradiol 953

oestrogen 1451

oestrogen metabolites 1458

oestrogen receptor *α* 1676

oestrogen-mimicking 441

oestrogens 709

oncolytic viruses 1512

Oncotype Dx 1342

one-step nucleic acid amplification assay 1197

oophorectomy 22

oral cancer knowledge 925

oral contraceptive use 1436

oral mucosa 1582

oral squamous cell carcinoma 1322

oral–oesophageal cancer 157

orthotopic model 1503

osteonectin 288

osteopenia 22

osteopontin 288, 542

osteoporosis 22

osteosarcoma 1503, 1839

outcome 139

ovarian cancer 304, 312, 360, 890, 989, 1436, 1593

ovarian carcinogenesis 1818

ovary 493, 723

overall survival 58, 1288, 1574

oxaliplatin 58

oxidative stress 469

p21^CIP1/WAF1^ 1210

p53 272, 469, 1012, 1593

p53 isoforms 1593

p73 1593

paclitaxel 897

paediatric 575, 1396

pancreatic cancer 523, 1554, 1733

pancreatic ductal adenocarcinoma 288

panitumumab 1495

parental occupational exposure 1409

PARP 1313

patient information 475

patupilone 1646

PEA3 124

PEDF 1503

pelvic 737

pelvic radiotherapy toxicity 903

persistence 694

personalised medicine 139, 246

PET imaging 1850

P-glycoprotein 513

PHA-665752 814

pharmacogenetics 53

Phase 1a trial 1830

phase I 1640

phase II 194, 599, 640, 1123

phosphatases 1235

photodynamic therapy 961, 1512

physical activity 1443

phyto-oestrogens 1151

PI3K 329

PI(3)K signalling pathway 281

PIF receptor 83

*PIK3CA* 281, 1920

PIM 1563

plasma 104, 239, 1733

plasminogen activator inhibitor 1 1750

platinum sensitivity 1144

pneumothorax 1912

podocalyxin-like 666

Poly (ADP-ribose) polymerases 1114

polymorphism 1244

pooled analysis 38, 890

population-based 1042, 1388, 1419

positive lymph nodes 1137

postmenopausal breast cancer prognosis 1151

postoperative complications 1279

post-traumatic growth 1158

*PPP1CA* 833

practice guideline 1166

preclinical 628

prediction 360

predictive 1487

predictive factor 9

predictive marker 255, 1693

premenopausal 1451

prevalence 38, 445

prevention 1776

primary care 475

primary cytoreduction 1818

prognosis 93, 304, 360, 666, 698, 842, 970, 975, 989, 1144, 1191, 1322, 1346, 1352, 1726, 1864, 1905, 1940

prognostic 1487

prognostic factor 89, 131

prognostic model 612

programme effectiveness 753

progression 1379

progression-free survival 1144

projections 1795

proliferation 552

prospective cohort 1061, 1430

prospective studies 157, 445

prospero-related homeobox gene 1346

prostate cancer 438, 481, 602, 847, 864, 931, 1061, 1230, 1362, 1600, 1628, 1640

prostate cancer diagnosis 65

prostatectomy 931

prostate-specific antigen 602

proteolysis-inducing factor (PIF) 83

proteosome 428

proton pump inhibitors 1173

PROX1 1346

PSC 1370

PTEN 1313, 1920

public education campaigns 925

pyrosequencing 246

qPCR 320

QResearch 452

qualitative research 753

quality of life 1158, 1495

quantitative evaluation 753

quantitative methylation test 65

quercetin 221

quiescence 1253

radiation therapy 534, 737

radiofrequency electromagnetic fields 640

radiotherapy 1628

randomised controlled trial 18, 945, 1905

randomised trials 1107

reactive oxygen species 1331

real-time RT–PCR 1600

receptor tyrosine kinase inhibition 649

rectal cancer 1654

recurrence 796, 890

recurrence-free survival 1487

recurrent ovarian cancer 1144

refractory 1635

regional ablation 931

registries 1042, 1069

regulatory T cells 413

relapsed ovarian cancer 884

relapse-free survival (RFS) 1574

renal cancer 112, 1912, 1811

renal cell carcinoma 1191, 1563, 1635, 1772

reovirus 1012

reproductive history 1436

resistance 796, 1131, 1322, 1331

response 682

review 13, 486

rib anomalies 1392

ribosome 329

risk 154, 881, 1396, 1776

risk factors 340

rural health 1039

S100A 263

S100A4 1379

S100beta 118

saracatinib 1030

sarcopenia 1469

scirrhous gastric carcinoma 996

screening 694, 983, 1388, 1669

screening interval 1082

SDF-1*α* 1615

second cancer 1076

seeds in diet 1151

segmental chromosome alterations 1940

self-sampling 337, 694

senescence 1235

sentinel lymph nodes 413

sentinel node biopsy 1197

serial testing, primer sequence 65

serine protease 1608

serology 1768

serum proteomic profiling 1370

sex hormone-binding globulin 709

sexual morbidity 903

sexually transmitted infections 602

SGI-1776 1563

SHP2 1235

silver *in situ* hybridisation 255

single-nucleotide polymorphism 870, 1676

skin cancer 1076

skin rash 938

skin toxicity 206, 1480

small-cell lung cancer 74, 746, 814

smoking 1096, 1430

SNAIL 393

SNPs 864

socio-economic status 1039, 1042, 1684, 1783

solid tumours 1402

somatic cancers 854

sorafenib 353, 1635

SP-B 673

spheroid invasion 565

splines 1795

squamous cell carcinoma 420, 618, 1582, 1608

SRC-1 118

staff development 340

stage 1039, 1042

STAT3 212, 407

stem cells 970, 1253

stomach neoplasms 413

stratification 1864

stroke 1419

stroma 1856

substaging 1137

sunitinib 1563, 1635

surgery 890, 1279

survival 170, 200, 420, 493, 542, 746, 890, 989, 1049, 1151, 1267, 1402, 1726, 1811, 1818, 1905

symptom awareness 1474

synergism 731

synthetic lethality 372

systematic review 194

tarceva 1850

targeted agents 1542

taxanes 1480

T-bet 366

Tcf-4 542

telomere 1772

tetraspanin 1002

TGF-*β* 996, 1856, 1885

therapy 682, 1030

thymidylate synthase 1542

thyroid 1396

time trade-off technique 606

time trends 723

tissue engineered 1582

TKI 1811

TMPRSS4 1608

TNM staging 842

transcription factors 312

transcriptional regulation 231

translational control 329

trans-phosphorylation 807

transplantation 970

trastuzumab 366, 796, 1176, 1273

trends 1414

triage 983

trial design 194

TTF-1 673

tumour ablation 1295

tumour cell content 89

tumour chemoimmunotherapy 1533

tumour immunology 413

tumour invasion 824

tumour marker 239, 552, 1741

tumour microenvironment 1224

tumour pattern 1818

tumour progression 1805

tumour suppressor 833

tumour vasculature 1512

tumourigenesis 523, 1856

tumour-infiltrating lymphocytes 93, 1191

tumour response 682

tumour-specific modulation frequencies 640

tumour-suppressor gene 74

turkey embryo xenograft 1708

type I and type II tumours 1818

tyrosine kinase inhibitor 1635

ubiquitin 428

UCP2 469

UGT1A1 53

UK 1082

uncoupling proteins 469

unifocal 931

urine 1894

uterine cancer 1137

uterine serous cancer 1176

Vγ9Vδ2 T cells 778

vaccination 486

vaccine 28

validation 1244

vandetanib 382

vascular endothelial growth factor 975

VE-cadherin 263

VEGFA 1856

viral hepatitis 1430

visual analogue scale 606

vitamin D_3_ 1874

volatile organic metabolites 1894

von Hippel-Lindau (VHL) disease 112

vulvar cancer 1279

willingness to pay 606

Wnt pathway inhibitors 1927

Wnt signalling 542

Wnt/*β-*catenin signalling 552

YB-1 1864

YKL-40 1203

yolk sac tumour 575

young women 177

ZEB1 263

